# Adjuvant immunotherapy improves recurrence-free and overall survival following surgical resection for intermediate/advanced hepatocellular carcinoma *a multicenter propensity matching analysis*

**DOI:** 10.3389/fimmu.2023.1322233

**Published:** 2024-01-08

**Authors:** Xiao Xu, Ming-Da Wang, Jia-Hao Xu, Zhong-Qi Fan, Yong-Kang Diao, Zhong Chen, Hang-Dong Jia, Fu-Bao Liu, Yong-Yi Zeng, Xian-Ming Wang, Han Wu, Wei Qiu, Chao Li, Timothy M. Pawlik, Wan Yee Lau, Feng Shen, Guo-Yue Lv, Tian Yang

**Affiliations:** ^1^ Department of Hepatobiliary and Pancreatic Surgery, General Surgery Center, First Hospital of Jilin University, Changchun, Jilin, China; ^2^ Department of Gastrointestinal Surgery, Wuhan Fourth Hospital, Wuhan, Hubei, China; ^3^ Department of Hepatobiliary Surgery, Eastern Hepatobiliary Surgery Hospital, Navy Medical University, Shanghai, China; ^4^ Department of Hepatobiliary Surgery, Affiliated Hospital of Nantong University, Nantong, Jiangsu, China; ^5^ Department of Hepatobiliary and Pancreatic Surgery, Zhejiang Provincial People’s Hospital, Hangzhou, Zhejiang, China; ^6^ Department of General Surgery, The First Affiliated Hospital of Anhui Medical University, Hefei, Anhui, China; ^7^ Department of Hepatobiliary Surgery, Mengchao Hepatobiliary Hospital, Fujian Medical University, Fuzhou, Fujian, China; ^8^ Department of General Surgery, The First Affiliated Hospital of Shandong First Medical University & Shandong Provincial Qianfoshan Hospital, Jinan, Shandong, China; ^9^ Department of Surgery, Ohio State University, Wexner Medical Center, Columbus, OH, United States; ^10^ Faculty of Medicine, The Chinese University of Hong Kong, Shatin, New Territories, Hong Kong SAR, China

**Keywords:** hepatocellular carcinoma, BCLC staging, recurrence, adjuvant therapy, immune checkpoint inhibitors, propensity matching analysis, recurrent-free survival, overall survival

## Abstract

**Background & aims:**

The effectiveness of adjuvant immunotherapy to diminish recurrence and improve long-term prognosis following curative-intent surgical resection for hepatocellular carcinoma (HCC) is of increased interest, especially among individuals at high risk of recurrence. The objective of the current study was to investigate the impact of adjuvant immunotherapy on long-term recurrence and survival after curative resection among patients with intermediate/advanced HCC.

**Methods:**

Using a prospectively-collected multicenter database, patients who underwent curative-intent resection for Barcelona Clinic Liver Cancer (BCLC) stage B/C HCC were identified. Propensity score matching (PSM) analysis was used to compare recurrence-free survival (RFS) and overall survival (OS) between patients treated with and without adjuvant immune checkpoint inhibitors (ICIs). Multivariate Cox-regression analysis further identified independent factors of RFS and OS.

**Results:**

Among the 627 enrolled patients, 109 patients (23.3%) received adjuvant immunotherapy. Most ICI-related adverse reactions were grading I-II. PSM analysis created 99 matched pairs of patients with comparable baseline characteristics between patients treated with and without adjuvant immunotherapy. In the PSM cohort, the median RFS (29.6 *vs.* 19.3 months, *P*=0.031) and OS (35.1 *vs.* 27.8 months, *P*=0.036) were better among patients who received adjuvant immunotherapy versus patients who did not. After adjustment for other confounding factors on multivariable analyzes, adjuvant immunotherapy remained independently associated with favorable RFS (HR: 0.630; 95% CI: 0.435-0.914; *P*=0.015) and OS (HR: 0.601; 95% CI: 0.401-0.898; *P*=0.013). Subgroup analyzes identified potentially prognostic benefits of adjuvant immunotherapy among patients with intermediate-stage and advanced-stage HCC.

**Conclusion:**

This real-world observational study demonstrated that adjuvant immunotherapy was associated with improved RFS and OS following curative-intent resection of intermediate/advanced HCC. Future randomized controlled trials are warranted to establish definitive evidence for this specific population at high risks of recurrence.

## Introduction

Hepatocellular carcinoma (HCC) poses a significant global health challenge, ranking as the sixth most prevalent cancer and the fourth leading cause of cancer-related death across the globe (1, 2). The Barcelona Clinic Liver Cancer (BCLC) staging system is a widely accepted tool for prognostic prediction and treatment allocation in HCC (3–5). This system stratifies HCC patients into five stages based on tumor burden, liver function, and patient performance status, recommending specific treatments for each stage including surgical resection, liver transplantation, local ablation, transarterial chemoembolization (TACE), or systemic therapy (3, 6). Despite the BCLC’s widespread use, management of intermediate/advanced HCC (BCLC stage B/C) remains contentious (7–12). Almost all Western guidelines caution against surgical resection for intermediate/advanced HCC (BCLC stage B/C) due to high morbidity and mortality associated with significant liver dysfunction and tumor burden, instead advocating for TACE or systemic therapy (4, 11, 13, 14). In the real world, surgical resection is still performed by surgeons worldwide, particularly in many Asian countries where the incidence of HCC is highest (9, 15–22). Many previous studies have revealed that long-term prognosis following surgical resection for intermediate/advanced HCC may be superior to TACE or systemic therapy (16, 19, 20, 23–28). The high rate of postoperative recurrence remains a significant bottleneck to improve surgical outcomes in this specific population (29–31). As such, there is an urgent need to explore and develop efficacious neoadjuvant and/or adjuvant therapies aimed at enhancing the long-term survival outcomes for this patient cohort who are at high risks of recurrence after resection.

Adjuvant immunotherapy, especially with immune checkpoint inhibitors (ICIs), has emerged as a promising approach to reduce recurrence and improve survival for various malignant tumors (32–39). ICIs represent a category of immunotherapeutic agents designed to selectively target immune regulatory pathways, thereby reinstating the anti-tumor immune response (40, 41). Pembrolizumab (42, 43), tirellizumab (44), sintilimab (45), camrelizumab (46), atezolizumab (47), tremelimumab plus durvalumab (48) have demonstrated efficacy in the management of advanced-stage HCC (41). However, the real-world effectiveness of adjuvant immunotherapy to enhance the oncological prognosis following curative-intent HCC resection, especially among patients at high risks of postoperative recurrence, remains to be elucidated.

The present study aims to explore the impact of adjuvant immunotherapy on long-term recurrence and survival after surgical resection for intermediate-/advanced-stage HCC. Utilizing a prospectively-collected multicenter database, propensity score matching (PSM) analysis was employed to compare recurrence-free survival (RFS) and overall survival (OS) between patients who did and did not receive postoperative adjuvant immunotherapy. The findings may provide valuable insights into the potential benefits of adjuvant immunotherapy in this high-risk population and guide future therapeutic strategies.

## Materials and methods

### Patient population

Using a prospectively collected multicenter database, patients who underwent curative-intent resection for intermediate/advanced (BCLC stage B/C) HCC from January 2018 to July 2022 at 7 Chinese hospitals (Eastern Hepatobiliary Surgery Hospital, First Hospital of Jilin University, the Affiliated Hospital of Nantong University, Zhejiang Provincial People’s Hospital, the First Affiliated Hospital of Anhui Medical University, Mengchao Hepatobiliary Hospital, and Qianfoshan Hospital of Shandong Province) were retrospectively identified. Patients were included in the analytic cohort who: 1) had pathologically confirmed HCC; 2) had intermediate/advanced HCC (BCLC stage B/C); 3) underwent curative-intent resection for HCC, which was defined as complete resection of all microscopic and macroscopic tumors (R0 resection), and with the first postoperative evaluation demonstrating absence of any residual or recurrent disease within 4~6 weeks after surgery. Patients were excluded who 1) were under 18 years of age; 2) had recurrent HCC; 3) had received preoperative anti-HCC treatments, including TACE, portal vein embolization, and systemic therapy (including chemotherapy, molecular targeted therapy, and immunotherapy); 4) had palliative liver resections, i.e. microscopically positive (R1 resection) or grossly positive (R2 resection) resection margins, or had residual or recurrent diseases at the first follow-up; 5) had adjuvant molecular targeted therapy after surgery; 6) were lost to follow-up within 90 days after surgery; and 7) had missing data on prognostic variables or follow-up information. Data in the study were censored on 31 December 2022. Written, informed consent for the data to be used for clinical research was obtained from all participating patients. This study was approved by the institutional review boards of all participating centers and conducted in accordance with the Declaration of Helsinki. Patient data were anonymized to ensure confidentiality.

### Data collection and variables

Patient demographics, clinical characteristics, laboratory findings, radiological and pathological features, surgical data, adjuvant ICIs medication usage, and follow-up data were prospectively collected from the medical records at each participating center, and retrospectively studied. The advantage of prospective data collection is that the data collected aligns more closely with the actual circumstances. The following variables were analyzed: age, sex, ECOG performance status, hepatitis B virus (HBV) infection status, Child-Pugh grading, presence of liver cirrhosis, serum alpha-fetoprotein (AFP) levels, maximum tumor size, tumor number, presence of macrovascular invasion, presence of microvascular invasion, presence of satellite nodules, tumor encapsulation, blood loss, transfusion, extent of liver resection, resection margins, adjuvant transarterial chemoembolization (TACE) and adjuvant immunotherapy. Minor liver resection was defined as resection of fewer than three Couinaud liver segments, while major liver resection was defined as resection of three or more liver segments. Non-anatomical liver resection included limited resection or wedge resection; anatomical resections were defined by the Brisbane 2000 system.

### Adjuvant immunotherapy

Patients with intermediate/advanced-stage HCC would be recommended for adjuvant immunotherapy (ICIs) and/or TACE 4~6 weeks after surgery, and the decision ultimately depended on the patient’s wishes. Adjuvant ICIs included pembrolizumab, sintilimab, camrelizumab, toripalimab, and tislelizumab, which were administered for 12 months according to the recommended dosages starting from 4~6 weeks after surgery until HCC recurrence serious adverse events, patient automagical withdrawal, death or occurrence of other conditions necessitating treatment termination. Generally, 3 weeks treatment was taken as one course, and patients in the adjuvant immunotherapy group received at least 3 months of treatment. The selection of ICI agents, dosage regimens, and treatment duration were determined by the attending physicians at each of the collaborating centers. Intermittent or reduced dosage was allowed during treatment to reduce drug-related toxicities. Adverse events were classified according to the National Cancer Institute Common Terminology Criteria for Adverse Events (CTCAE) version 5.0. The use of adjuvant ICI treatment and immune-related adverse events (irAEs) were recorded.

### Study endpoints

The primary endpoints of this study were RFS and OS. RFS was defined as the time from surgery to the detection of recurrence, death from any cause, or the last follow-up, whichever occurred first, while OS was defined as the time from surgery to death from any cause or the last follow-up. The secondary endpoint was the incidence of irAEs among patients who received adjuvant immunotherapy.

### Propensity score matching (PSM) analysis

To mitigate potential biases and confounding factors in the comparative analysis of outcomes between the groups with and without adjuvant immunotherapy, a rigorous PSM analysis was conducted (1, 49–51). The propensity scores were calculated using a logistic regression model, based on the following covariates: age, sex, ECOG performance status, HBV infection status, Child-Pugh grading, liver cirrhosis, preoperative serum AFP levels, largest tumor diameter, tumor number, macrovascular invasion, microvascular invasion, satellite nodules, tumor encapsulation, intraoperative blood loss, intraoperative blood transfusion, extent of hepatectomy, and adjuvant TACE. Patients in both the adjuvant immunotherapy and non-adjuvant immunotherapy groups were meticulously matched in a 1:1 ratio using the nearest neighbor matching method, with a caliper width set at 0.05 times the standard deviation of the logit of the propensity score to ensure optimal comparability. The balance of the matched variables between the two groups was assessed using standardized mean differences (SMD), with an SMD of less than 0.2 indicating a negligible difference in the mean or prevalence of the covariates between the matched groups.

### Statistical analysis

Descriptive statistics were employed to concisely summarize the baseline characteristics of the patients involved in this study. Continuous variables were reported as means with standard deviations (SD) or medians with interquartile ranges (IQR), as appropriate. Categorical variables were expressed as frequencies and percentages. The comparisons of continuous variables between groups were performed using the independent t-test or the Mann-Whitney U test, as appropriate. The chi-square test or Fisher’s exact test was used to compare categorical variables. Kaplan-Meier survival analysis was used to estimate RFS and OS, and the log-rank test was applied to compare the survival differences between the groups with and without adjuvant immunotherapy. Univariate and multivariate Cox proportional hazards regression models were used to identify the independent prognostic factors associated with RFS and OS. Variables with a *P*-value less than 0.10 in the univariate analysis were included in the multivariate analysis. The results were presented as hazard ratios (HR) with 95% confidence intervals (CI). Subgroup analyzes in the PSM cohort were performed to identify the potential prognostic benefit of adjuvant immunotherapy among patients with intermediate-stage and patients with advanced-stage HCC, respectively. All statistical analyzes were performed using the R software version 4.0.3 (R Foundation for Statistical Computing, Vienna, Austria) and SPSS version 26.0 (IBM Corp., Armonk, NY, USA). A two-sided P-value less than 0.05 was considered statistically significant.

## Results

### Patient characteristics

A total of 1542 HCC patients were screened for eligibility, of which 627 patients with intermediate/advanced-stage HCC (BCLC stage B/C) met the inclusion criteria (**Supplementary Figure 1**). Among these individuals, 109 patients received adjuvant immunotherapy treatment (the adjuvant immunotherapy group) and 518 patients did not receive adjuvant immunotherapy treatment (the non-adjuvant immunotherapy group). PSM created 99 patient pairs who received and did not receive adjuvant immunotherapy. Patient clinical characteristics and operative variables between the two groups in the entire and PSM cohorts are shown in **Table 1**.

**Table 1:** Comparison of patient baseline characteristics and operative variables in the entire and PSM cohorts.

n (%)	The entire cohort				The PSM cohort			
	With adjuvant Immunotherapy (n =109)	Without adjuvant Immunotherapy (n = 518)	*P*	*SMD*	With adjuvant Immunotherapy (n = 99)	Without adjuvant Immunotherapy (n = 99)	*P*	*SMD*
Age, years*	56.7 ± 12	56.2 ± 11	0.680	0.043	56.5 ± 12	56.7 ± 11	0.915	0.015
Male sex	93 (85.3)	439 (84.7)	0.996	0.016	84 (84.8)	87 (87.9)	0.679	0.088
ECOG performance status 1-2	33 (30.3)	244 (47.1)	0.002	0.351	32 (32.3)	40 (40.4)	0.301	0.169
HBV (+)	82 (75.2)	427 (82.4)	0.107	0.177	74 (74.7)	77 (77.8)	0.738	0.071
Child-Pugh grade B	9 (8.3)	70 (13.5)	0.179	0.169	9 (9.1)	7 (7.1)	0.794	0.074
Cirrhosis	65 (59.6)	483 (73.9)	0.004	0.307	63 (63.6)	58 (58.6)	0.560	0.104
AFP level > 400 ug/L	40 (36.7)	198 (38.2)	0.849	0.032	36 (36.4)	36 (36.4)	1.000	< 0.001
Maximum tumor size > 5 cm	66 (60.6)	295 (56.9)	0.559	0.073	62 (62.6)	60 (60.6)	0.884	0.042
Multiple tumors	38 (34.9)	170 (32.8)	0.764	0.043	38 (38.4)	40 (40.4)	0.884	0.041
Macrovascular invasion	9 (8.3)	81 (15.6)	0.065	0.229	22 (22.2)	19 (19.2)	0.726	0.075
Microvascular invasion	35 (32.1)	274 (52.9)	< 0.001	0.430	33 (33.3)	27 (27.3)	0.439	0.132
Satellite nodules	33 (30.3)	91 (17.6)	0.004	0.301	27 (27.3)	32 (32.3)	0.534	0.111
Incomplete tumor encapsulation	85 (78.0)	346 (66.8)	0.030	0.252	77 (77.8)	81 (81.8)	0.595	0.101
Intraoperative blood loss > 400 ml	86 (78.9)	305 (58.9)	< 0.001	0.443	23 (23.2)	24 (24.2)	1.000	0.024
Intraoperative blood transfusion	24 (22.0)	129 (24.9)	0.607	0.068	21 (21.2)	16 (16.2)	0.466	0.130
Major hepatectomy	31 (28.4)	189 (36.5)	0.136	0.172	28 (28.3)	36 (36.4)	0.287	0.173
Postoperative TACE	37 (33.9)	225 (43.4)	0.086	0.196	35 (35.4)	28 (28.3)	0.360	0.152

AFP, alpha-fetoprotein; ECOG, Eastern Cooperative Oncology Group; HBV, hepatitis B virus; PSM, propensity score matching; TACE, transarterial chemoembolization; IQR, interquartile range.

* Values are mean ± standard deviation.

**Figure 1:**
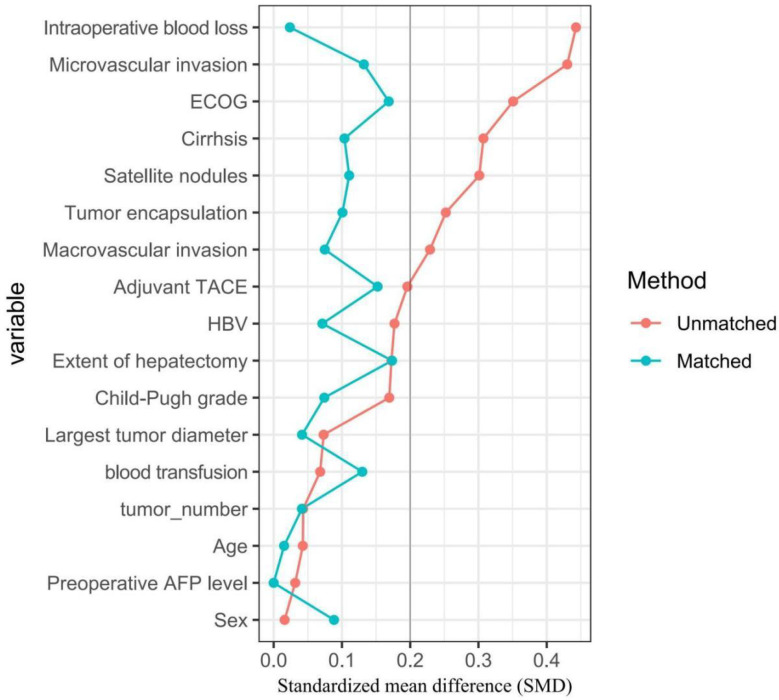
Comparisons of standardized mean difference of clinical variables between patients with and without adjuvant immunotherapy in the entire cohort (red dots), in the PSM cohort (green dots), respectively.

Compared with individuals who did not receive adjuvant immunotherapy, patients who received adjuvant immunotherapy had a lower proportion of performance status 1-2 (30.3% *vs.* 47.1%, *P* = 0.002), cirrhosis (59.6% *vs.* 73.9%, *P* = 0.004), and microvascular invasion (32.1% *vs.* 52.9%, *P* < 0.001), yet a higher proportion of satellite nodules (30.3% *vs.* 17.6%, *P* = 0.004), incomplete tumor encapsulation (78.0% *vs.* 66.8%, *P* = 0.030), and intraoperative blood transfusion > 400 ml (78.9% *vs.* 58.9%, *P* < 0.001). Of note, there were no significant differences among patients who did versus those who did not receive adjuvant immunotherapy for any covariate after matching (all *P* > 0.05, *SMD* < 0.2) (**Figure 1**).

### Adjuvant immunotherapy and adverse events

The ICI agents used among patients who received adjuvant immunotherapy (n=109) were tislelizumab (51.4%, n=56), sintilimab (29.3%, n=32), camrelizumab (9.2%, n=10), pembrolizumab (6.4%, n=7), and toripalimab (3.7%, n=4). All patients receiving adjuvant immunotherapy completed at least 3 months of ICI treatment, with no patients discontinuing the treatment due to irAEs. The overall incidence of irAEs among patients in the adjuvant immunotherapy group was 93.6% (99/109), and the most common irAEs were anorexia (84.8%), followed by fatigue (64.2%), allergic reactions (50.4%), gastrointestinal symptoms (e.g., vomiting or diarrhea; 44.0%), liver dysfunction (22.9%), proteinuria (7.3%), and anemia (5.5%). All of these irAEs were grade I-II, being manageable, and reversible with appropriate interventions, including dose adjustment, symptomatic treatment, or temporary discontinuation of ICI therapy.

### Survival outcomes in the entire and PSM cohorts

**Figure 2:**
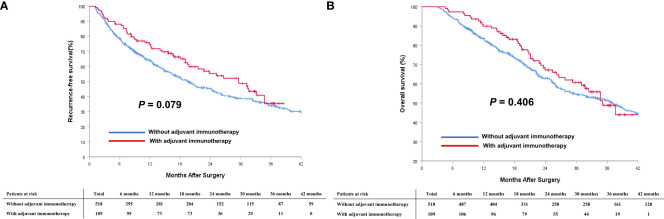
Comparisons of cumulative incidence of recurrence-free survival (RFS, **A**) and overall survival (OS, **B**) curves between patients with and without adjuvant immunotherapy in the entire cohort.

Comparison of survival outcomes among patients who did and did not receive adjuvant immunotherapy in the entire and PSM cohorts are noted in **Table 2**. In the entire cohort, the median RFS for patients who received adjuvant immunotherapy was not significantly better than that for patients who did not receive (29.6 months [95% CI 22.4 to 36.8 months] *vs.* 19.4 months [95% CI 16.0 to 22.8 months]; *P* = 0.079, **Figure 2A**); the median OS was also comparable (35.1 months [95% CI 29.9 to 40.3 months] *vs.* 37.1 months [95% CI 31.3 to 42.9 months]; *P* = 0.406, **Figure 2B**).

**Table 2:** Comparisons of oncological outcomes in the entire and PSM cohorts.

N (%)	The entire cohort			The PSM cohort		
	With adjuvant Immunotherapy (n = 109)	Without adjuvant Immunotherapy (n = 518)	*P*	With adjuvant Immunotherapy (n = 99)	Without adjuvant Immunotherapy (n = 99)	*P*
Period of follow-up, months*	24.5 (17.0-33.7)	24.0 (12.9-40.0)	0.693	24.6 (17.1-34.1)	27.8 (16.1-36.8)	0.371
Recurrence during the follow-up	53 (48.6)	309 (59.7)	0.034	49 (50.0)	69 (69.7)	0.004
Mortality during the follow-up	44 (40.4)	265 (51.2)	0.041	40 (40.0)	59 (60.0)	<0.001
Median RFS, 95% CI, months	29.6 (22.4-36.8)	19.4 (16.0-22.8)	0.079	29.6 (21.7-37.5)	19.3 (13.1-25.5)	0.031
1-year RFS, %	74.9	63.9		74.3	59.3	
2-year RFS, %	55.3	45.3		56.3	41.3	
3-year RFS, %	35.4	33.7		35.1	25.3	
Median OS, 95% CI, months	35.1 (29.9-40.3)	37.1 (31.3-42.9)	0.406	35.1 (29.3-40.9)	27.8 (22.6-33.0)	0.036
1-year OS, %	93.7	83.7		88.8	84.8	
2-year OS, %	80.9	62.9		67.8	56.6	
3-year OS, %	51.5	58.6		50.0	36.9	

CI, confidence interval; RFS, recurrence-free survival; OS, overall survival; PSM, propensity score matching.

* Values are median and range.

**Figure 3:**
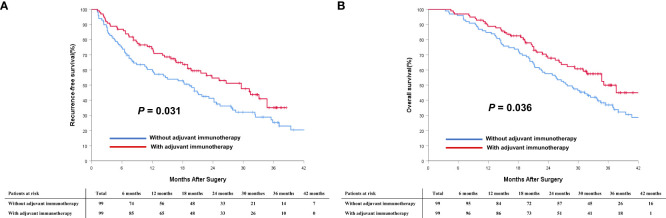
Comparisons of cumulative incidence of recurrence-free survival (RFS, **A**) and overall survival (OS, **B**) curves between patients with and without adjuvant immunotherapy in the PSM cohort.

In the PSM cohort, the median RFS for patients who received adjuvant immunotherapy was significantly longer than those patients who did not receive adjuvant immunotherapy (29.6 months [95% CI 21.7 to 37.5] *vs.* 19.3 months [95% CI 13.1 to 25.5]; *P* < 0.001, **Figure 3A**). Similarly, the median OS was significantly more favorable in the adjuvant immunotherapy group than in the non-adjuvant immunotherapy group (35.1 months [95% CI 29.3 to 40.9] *vs.* 27.8 months [95% CI 22.6 to 33.0]; *P* < 0.001, **Figure 3B**).

### Univariate and multivariate analysis of RFS and OS in the PSM cohort

Univariate and multivariate Cox-regression analyzes to identify independent factors associated with RFS and OS in the PSM cohort are shown in **Tables 3**, **4**, respectively. On multivariate analysis, after adjusting for other confounding factors, postoperative adjuvant immunotherapy remained independently associated with more favorable RFS (HR: 0.630, 95% CI 0.435-0.914, *P* = 0.015) and OS (HR: 0.601, 95% CI 0.401-0.898, *P* = 0.013) after surgical resection for intermediate/advanced-stage HCC.

**Table 3:** Univariate and multivariate Cox-regression analyzes predicting recurrence-free survival in the PSM cohort.

Variables	HR comparison	UV HR (95% CI)	UV *P*	MV HR (95% CI)	MV *P*
Age	≤ 60 years *vs.* > 60 years	0.795 (0.547-1.156)	0.230		
Sex	Male *vs.* female	0.859 (0.491-1.504)	0.595		
ECOG performance status	1-2 *vs.* 0	1.292 (0.893-1.868)	0.174		
HBV (+)	Yes *vs.* no	1.817 (1.149-2.875)	0.105		
Child-Pugh grade	B *vs.* A	1.388 (0.724-2.661)	0.324		
Cirrhosis	Yes *vs.* no	1.034 (0.715-1.494)	0.860		
Preoperative AFP level	> 400 *vs.* ≤ 400 ug/ml	1.083 (0.744-1.576)	0.679		
Largest tumor diameter	≤ 5 cm *vs.* > 5 cm	1.877 (1.263-2.789)	0.002	1.837 (1.194-2.824)	0.006
Tumor number	Multiple *vs.* solitary	2.233 (1.550-3.216)	< 0.001	2.455 (1.673-3.602)	< 0.001
Macrovascular invasion	Yes *vs.* no	2.086 (1.373-3.171)	0.001	NS	0.080
Microvascular invasion	Yes *vs.* no	1.809 (1.237-2.645)	0.002	1.585 (1.054-2.382)	0.027
Satellite nodules	Yes *vs.* no	1.473 (1.005-2.158)	0.047	NS	0.802
Tumor encapsulation	Incomplete *vs.* complete	0.903 (0.557-1.464)	0.679		
Intraoperative blood loss	> 400 *vs.* ≤ 400 ml	0.811 (0.521-1.262)	0.353		
Intraoperative blood transfusion	Yes *vs.* no	0.666 (0.393-1.130)	0.132		
Extent of hepatectomy	Major *vs.* minor	0.859 (0.584-1.264)	0.441		
Adjuvant TACE	Yes *vs.* no	1.361 (0.928-1.994)	0.114		
Adjuvant immunotherapy	Yes *vs.* no	0.669 (0.463-0.967)	0.032	0.630 (0.435-0.914)	0.015

AFP, alpha-fetoprotein; CI, confidence interval; ECOG, Eastern Cooperative Oncology Group; HBV, hepatitis B virus; HR, hazard ratio; MV, multivariate, NS, not significant; TACE, transarterial chemoembolization.

**Table 4:** Univariate and multivariate Cox-regression analyzes predicting overall survival in the PSM cohort.

Variables	HR comparison	UV HR (95% CI)	UV *P*	MV HR (95% CI)	MV *P*
Age	≤ 60 years *vs.* > 60 years	0.879 (0.584-1.300)	0.517		
Sex	Male *vs.* female	1.378 (0.829-2.290)	0.216		
ECOG performance status	1-2 *vs.* 0	0.893 (0.601-1.328)	0.578		
HBV (+)	Yes *vs.* no	1.294 (0.821-2.040)	0.267		
Child-Pugh grade	B *vs.* A	0.911 (0.423-1.963)	0.811		
Cirrhosis	Yes *vs.* no	0.979 (0.667-1.438)	0.915		
Preoperative AFP level	> 400 *vs.* ≤ 400 ug/ml	1.174 (0.797-1.728)	0.416		
Largest tumor diameter	≤ 5 cm *vs.* > 5 cm	1.587 (1.052-2.393)	0.028	NS	0.208
Tumor number	Multiple *vs.* solitary	1.535 (1.049-2.246)	0.027	1.532 (1.032-2.273)	0.034
Macrovascular invasion	Yes *vs.* no	2.689 (1.783-4.056)	<0.001	2.422 (1.507-3.893)	<0.001
Microvascular invasion	Yes *vs.* no	1.448 (0.970-2.162)	0.070	NS	0.660
Satellite nodules	Yes *vs.* no	1.577 (1.059-2.351)	0.025	NS	0.920
Tumor encapsulation	Incomplete *vs.* complete	1.522 (0.970-2.388)	0.068	1.790 (1.117-2.867)	0.015
Intraoperative blood loss	> 400 *vs.* ≤ 400 ml	0.894 (0.567-1.411)	0.631		
Intraoperative blood transfusion	Yes *vs.* no	1.330 (0.830-2.129)	0.235		
Extent of liver resection	Major *vs.* minor	0.858 (0.573-1.286)	0.459		
Adjuvant TACE	Yes *vs.* no	1.137 (0.593-2.180)	0.699		
Adjuvant immunotherapy	Yes *vs.* no	0.657 (0.443-0.975)	0.037	0.601 (0.401-0.898)	0.013

AFP, alpha-fetoprotein; CI, confidence interval; ECOG, Eastern Cooperative Oncology Group; HBV, hepatitis B virus; HR, hazard ratio; MV, multivariate, NS, not significant; TACE, transarterial chemoembolization.

### Subgroup analysis

**Figure 4:**
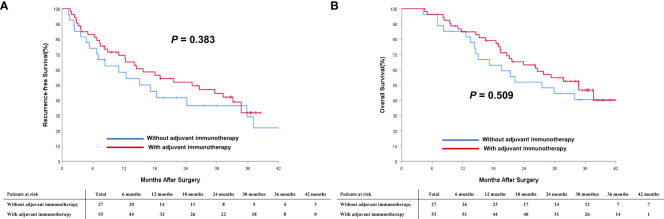
Comparisons of cumulative incidence of recurrence-free survival (RFS, **A**) and overall survival curves (OS, **B**) between patients with and without adjuvant immunotherapy in the BCLC B stage cohort.

**Figure 5:**
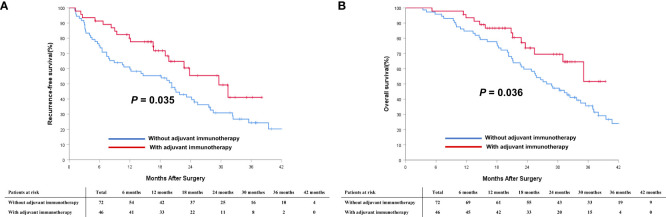
Comparisons of cumulative incidence of recurrence-free survival (RFS, **A**) and overall survival curves (OS, **B**) between patients with and without adjuvant immunotherapy in the BCLC C stage cohort.

To understand better the potential effectiveness of adjuvant immunotherapy, analyzes stratified by different BCLC tumor stages, subgroup analyzes of patients with intermediate-stage (BCLC stage B) HCC and advanced-stage (BCLC stage C) HCC were performed. In the PSM cohort, 53 (66.3%) patients with intermediate-stage HCC and 46 (39.0%) with advanced-stage HCC received adjuvant immunotherapy after surgery. As shown in **Figure 4**, in the cohort of patients with intermediate-stage HCC, there was a trend toward better RFS among patients who received adjuvant immunotherapy versus individuals who did not receive adjuvant immunotherapy (2-year RFS rates: 41.5% *vs.* 29.6%; *P* = 0.383). There was a trend toward better OS among patients who received adjuvant immunotherapy versus individuals who did not receive adjuvant immunotherapy (2-year OS rates: 58.5% *vs.* 51.9%; *P* = 0.509). A similar trend was noted in the cohort of patients with advanced-stage HCC (**Figure 5**). There was a trend toward better RFS among patients who did versus those did not receive adjuvant immunotherapy (2-year RFS rates: 34.7% *vs.* 23.9%; *P* = 0.035). There was a trend toward better OS among patients who received adjuvant immunotherapy versus those who did not receive adjuvant immunotherapy (2-year OS rates: 59.7% *vs.* 43.5%; *P* = 0.036).

## Discussion

Nevertheless, even in patients who are considered to be ideal candidates for curative treatment, the recurrence rates following resection or ablation have been documented to exceed 70% within five years (52). Furthermore, in patients with more advanced tumor burden undergoing curative-intent therapies, the risk of HCC recurrence is further amplified (53, 54). The present study focused on patients with intermediate and advanced-stage HCC who underwent curative-intent surgical resection, and attempted to highlight the potential benefits of adjuvant immunotherapy in a cohort of patients that Western guidelines might consider non-operable. This study is among the first to specifically investigate the impact of adjuvant immunotherapy in a real-world setting for HCC patients who are at high risks of tumor recurrence after curative-intent resection. Utilizing PSM analysis, the results of this multicenter real-world study provided aditional evidence to support the use of adjuvant immunotherapy in enhancing RFS and OS following curative-intent resection of intermediate-/advanced-stage HCC. These findings align with the growing body of literature that underscores the potential of adjuvant immunotherapy in the management of various malignancies (32, 34–37).

In the present study, adjuvant immunotherapy significantly improved RFS and OS after curative-intent surgical resection for intermediate/advanced-stage HCC. These findings are in agreement with outcomes obtained from contemporary clinical trials seeking to evaluate the therapeutic effectiveness of adjuvant immunotherapy among individuals diagnosed with HCC. The conclusive results of the IMbrave 050 trial have demonstrated that the combination of atezolizumab and bevacizumab demonstrated improved recurrence-free survival compared to active surveillance in patients with a high risk of HCC recurrence after curative-intent resection or ablation. To the best of our knowledge, the IMbrave 050 study is the first phase III trial evaluating adjuvant treatment for HCC to report positive outcomes. However, longer follow-up is still needed to comprehensively assess the survival benefit as well as potential risks of this regimen, particularly in terms of both recurrence-free and overall survival (55, 56).

In patients with HCC, the immune microenvironment manifests a pronounced abundance of immune cell infiltration, encompassing T cells, natural killer cells, macrophages, and dendritic cells (57–59). The PD-1/PD-L1 axis plays a crucial role in tumor immune evasion by suppressing the immune response (60–63). ICIs, by blocking the PD-1/PD-L1 axis, can restore the antitumor immune response and potentially eliminate residual tumor cells after curative HCC resection (64). Thus, there is an inherent theoretical advantage to employ adjuvant immunotherapy in HCC.

The notable irAEs observed in the present study, such as anorexia, fatigue, and allergic reactions, were aligned with known side effects of ICIs therapy (65). Fortunately, these adverse events were manageable and did not necessitate treatment discontinuation in any patients with adjuvant immunotherapy, underscoring the relative safety of these agents when monitored closely. Additionally, the subgroup analyzes further defined benefits of adjuvant immunotherapy across different BCLC tumor stages. While patients with intermediate-stage HCC was associated with a positive trend toward improved RFS and OS, patients with advanced-stage HCC derived the most benefit from ICI adjuvant therapy. This observation suggested that advanced-stage disease might harbor a more immunosuppressive microenvironment, which, when counteracted by ICIs, leads to a profound clinical response (62, 66–68).

While generally considered as the gold standard in clinical research, RCTs often have stringent inclusion and exclusion criteria, which may limit their generalizability to a broader patient population (69). Real-world studies, on the other hand, offer insights into the effectiveness of interventions in routine clinical practice, encompassing a more diverse patient population and may reflect more genuine clinical scenarios (70). For a considerable subset of HCC patients, especially in Asian contexts, who might find themselves at the crossroads of palliative care and aggressive intervention, our study offers a glimmer of hope. Not only do the data validate the decision for surgical resection, but also highlights the potential importance of adjuvant therapy for improving long-term outcomes. Moreover, multicenter studies, by virtue of their design, capture variations in practice patterns across different institutions, further enhancing the external validity of the findings. In addition, the use of PSM was to minimize the potential confounding effects inherent in observational studies. PSM ensures that the treated and untreated groups are balanced on observed covariates, thereby approximating the conditions of an RCT (50). This methodological approach strengthens the internal validity of our findings.

The current study has several limitations. As with all observational studies, there remains the potential for unmeasured confounding. While PSM can balance observed covariates, it cannot account for unobserved or unmeasured variables. In addition, the duration of follow-up in this study may not be sufficient to capture long-term outcomes and late complications associated with adjuvant immunotherapy. The heterogeneity in the types and regimens of ICIs used across different centers might introduce variability in outcomes. Of note, due to the wide range of PD-1/PD-L1 categories employed in our study and the small sample size, the number of patients receiving specific immunotherapy regimen within the subgroup analysis was relatively limited, leading to non-significant statistical results for subgroup analysis based on different drug types. Finally, the real-world nature of our study highly suggests that the decision to administer adjuvant immunotherapy was clinician-driven, potentially introducing selection bias. Our focus, while innovative, also necessitates multicenter validations, preferably through randomized controlled trials to further solidify the evidence. Future endeavors should aim to address these limitations by incorporating larger patient cohorts, diversified across various regions, and possibly introducing prospective study designs with using a single, standard immunotherapy regimen. Further explorations into the specific mechanisms by which adjuvant immunotherapy offers benefits in postoperative HCC patients could also pave the way for personalized therapeutic strategies.

In conclusion, this multicenter real-world PSM analysis provides promising evidence supporting the role of adjuvant immunotherapy to improve RFS and OS among patients with intermediate-/advanced-stage HCC who underwent curative-intent surgical resection. The findings underscore the potential of ICIs to enhance long-term outcomes in this specific population at high risks of recurrence. While our study offers valuable insights, future RCTs are essential to establish definitive evidence and further elucidate the optimal timing, duration, and regimen of adjuvant immunotherapy for HCC patients.

## Data availability statement

The raw data supporting the conclusions of this article will be made available by the authors, without undue reservation.

## Ethics statement

Data in the study were censored on 31 December 2022. Written, informed consent for the data to be used for clinical research was obtained from all participating patients. This study was approved by the institutional review boards of all participating centers and conducted in accordance with the Declaration of Helsinki. Patient data were anonymized to ensure confidentiality. The studies were conducted in accordance with the local legislation and institutional requirements. The participants provided their written informed consent to participate in this study. Written informed consent was obtained from the individual(s) for the publication of any potentially identifiable images or data included in this article.

## Author contributions

XX: Methodology, Visualization, Conceptualization, Data curation, Formal analysis, Writing – original draft, Investigation. M-DW: Funding acquisition, Resources, Conceptualization, Data curation, Formal analysis, Methodology, Writing – original draft. J-HX: Validation, Conceptualization, Data curation, Writing – original draft, Formal analysis. Z-QF: Conceptualization, Data curation, Writing – original draft, Formal analysis. Y-KD: Methodology, Software, Conceptualization, Data curation, Writing – original draft. ZC: Supervision, Writing – review & editing, Conceptualization, Data curation. H-DJ: Resources, Writing – original draft, Software, Conceptualization, Data curation. F-BL: Conceptualization, Data curation, Resources, Writing – original draft. Y-YZ: Funding acquisition, Supervision, Conceptualization, Data curation, Resources, Writing – original draft. X-MW: Methodology, Project administration, Conceptualization, Data curation, Writing – original draft. HW: Formal analysis, Validation, Conceptualization, Data curation, Writing – original draft. WQ: Resources, Software, Conceptualization, Data curation, Writing – original draft. CL: Conceptualization, Data curation, Writing – original draft. TP: Formal analysis, Supervision, Writing – review & editing, Conceptualization. WL: Writing – review & editing, Visualization, Conceptualization, Supervision. FS: Data curation, Conceptualization, Formal analysis, Supervision, Writing – review & editing. G-YL: Conceptualization, Data curation, Formal analysis, Supervision, Writing – review & editing. TY: Funding acquisition, Project administration, Writing – original draft, Conceptualization, Data curation, Formal analysis, Supervision, Writing – review & editing.

## Supplementary material

Supplementary Figure 1 Flow chart of participant population. HCC, hepatocellular carcinoma.
