# Supplementary material for: Adjuvant immunotherapy improves recurrence-free and overall survival following surgical resection for intermediate/advanced hepatocellular carcinoma a multicenter propensity matching analysis
Source: Front Immunol. 2024 Jan 8;14:1322233. doi: 10.3389/fimmu.2023.1322233 (PMC10806403; doi:10.3389/fimmu.2023.1322233)

**SUPPLEMENT FIGURE 1.** Flow chart of participant population. HCC, hepatocellular carcinoma.

Patients who underwent curative-intent hepatectomy for HCC at 9 Chinese hospitals from 2018 to 2022 (**N = 1542**)

**Exclusion (N = 915)**

≤ 18 years old (n = 16)

With distant metastasis (n = 90)

Preoperative tumor rupture (n = 37)

Recurrence HCC (n = 183)

Receiving [neoadjuvant](javascript:;) treatment before hepatectomy (n = 83)

Receiving adjuvant targeted therapy after hepatectomy (n = 40)

Non-curative resection (n = 53)

Postoperative 90-day death (n = 11)

Early-stage HCC (BCLC stage 0/A) (n = 342)

Loss to follow-up within 6 months after hepatectomy (n = 29)

Missing data on important prognostic variables (n = 31)

**Inclusion** **in the entire cohort** **(N = 627)**

With adjuvant immunotherapy (n = 109)

Without adjuvant immunotherapy (n = 518)

**Exclusion (N = 429)**

With adjuvant immunotherapy (n = 10)

Without adjuvant immunotherapy (n = 419)

**Inclusion in the PSM cohort (N = 198)**

With adjuvant immunotherapy (n = 99)

Without adjuvant immunotherapy (n = 99)

**1:1 PSM**

**SUPPLEMENT FIGURE 2.** Graphic Abstract


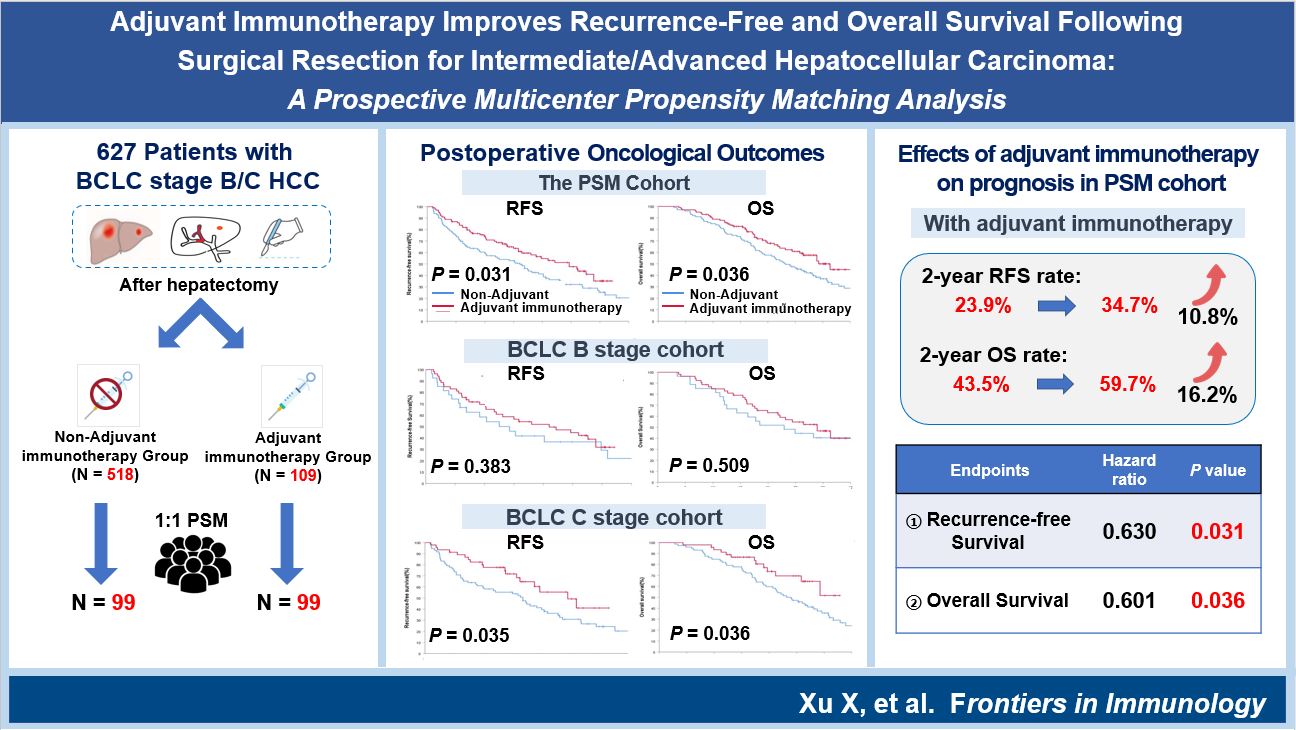

Supplement: Supplementary Figure 1 — Flow chart of participant population. HCC, hepatocellular carcinoma. [file DataSheet_1.docx]
